# Exploring the Diagnostic Dilemma of Indeterminate Pulmonary Nodules in Patients with Primary Sarcoma of Bone

**DOI:** 10.1155/2024/9926675

**Published:** 2024-03-05

**Authors:** Babe Westlake, Jeffrey Brown, Jacqueline Hart, Cameron Skiby, Kevin Jones, John Groundland

**Affiliations:** Department of Orthopaedics, Huntsman Cancer Institute, University of Utah, Salt Lake City, Utah, USA

## Abstract

**Introduction:**

Bone sarcomas are known to have a predilection for pulmonary metastasis. Surveillance protocols are thus focused on periodic chest imaging, typically with CT scan. Pulmonary nodules can be easily identified with this modality, but smaller nodules are not readily biopsied and may not represent metastatic disease. These are called indeterminate. The natural history of indeterminate nodules in a bone sarcoma population and factors associated with progression to true metastatic disease are not clearly defined.

**Methods:**

All bone sarcoma patients treated at a single institution from 2010 to 2020 were eligible for inclusion. We treated 327 patients over this period; 119 were excluded for age less than 16 years, 31 were excluded for evident metastatic disease at presentation, and 60 were excluded for incomplete clinical follow-up or CT chest imaging either at staging or in surveillance. We assessed chest CT images for presence of pulmonary nodules and selected variables both at the staging and on surveillance images. Nodules were considered metastatic if proven histologically with a biopsy or by clinical interpretation by the multidisciplinary sarcoma team. Clinical and imaging factors were assessed for the association of indeterminate nodule progression to true metastatic disease.

**Results:**

Seventy three of the 117 patients had indeterminate nodules on their staging CT scan; 41.1% of those patients progressed to metastatic disease compared to 43.2% of the patients that did not have indeterminate nodules on staging CT. Fifty eight of the 117 patients developed indeterminate nodules on surveillance chest CT, and 55.2% of those patients progressed to metastatic disease. There were no clinical or imaging factors that predicted the development of metastatic disease in the group that had indeterminate nodules at presentation; however, the number and size of nodules did correlate with progression to metastasis in those that developed indeterminate nodules on surveillance.

**Conclusion:**

Indeterminate pulmonary nodules are common on staging CT scans in patients with a bone sarcoma. The presence or absence of these indeterminate nodules was not predictive of progression to true metastatic disease in this cohort. However, the development of indeterminate nodules on surveillance imaging was associated with progression to metastatic disease with the size and number of nodules being important factors.

## 1. Introduction

Primary sarcomas of bone are a group of malignancies that originate from mesenchymal tissues and share a proclivity for pulmonary metastasis. While the different subtypes of bone sarcoma demonstrate slightly different rates of metastasis based on variables such as histology, grade, and anatomic location, the vast majority of all bone sarcoma patients who develop metastatic disease will have demonstrable disease in the lungs to the extent that it is estimated that over 90% of bone sarcoma patients who develop metastatic disease will have sites of metastasis in the lungs [1, 2]. Particularly troublesome, metastatic disease may present across a long period of time, ranging from the time of initial diagnosis to more than ten years after treatment. This is critical in the care and management of the sarcoma patient due to the fact that the presence of lung metastases has a profound impact on prognosis [3]. It is for these reasons that the imaging of the chest is a part of the recommended standard practice in the initial staging and the post-treatment surveillance of all patients with a primary sarcoma of bone.

In their recommendations for the initial staging of patients presenting with most primary sarcomas of bone, the National Comprehensive Cancer Network (NCCN) recommends imaging of the chest with computed tomography (CT chest) as an integral part of the initial workup of the patient [4]. CT scans of the chest can identify nodules as small as 2 mm, while biopsy of a pulmonary nodule is typically unreliable if the lesion is under 10 mm [5]. This splits sarcoma patients into several categories based on their chest CT scans as follows: (1) those with no evidence of pulmonary metastasis on CT; (2) those with pulmonary nodules measuring 2–9 mm, making them indeterminant in nature, as they can be identified by imaging but not reliably biopsied; and (3) those with nodules 10 mm or greater, which can be biopsied to potentially render a diagnosis of metastatic disease versus unrelated disease. Once the baseline CT chest is done, future scans can determine whether any new nodules develop or if an existing nodule grows or regresses.

In post-treatment surveillance for disease recurrence and metastasis, the NCCN recommendations include imaging of the chest with CT or radiographs, depending on the clinician's concern for the development of metastatic sites, based on the totality of the presentation of the sarcoma [4]. For high-grade sarcomas of bone, this often results in a schedule of CT scans of the chest, declining in frequency, over the period of 5–10 years. Findings on these scans place patients into similar categories as noted above: those with no nodules; those with nodules that are present on CT but unable to be biopsied; and those with nodules that can be biopsied to render a tissue diagnosis.

The rationale for early and then repeated imaging of the chest is to identify metastatic disease as early as possible so that treatments such as chemotherapy, radiation, or surgical resection may be given or adjusted. Likewise, negative chest scans are potentially very reassuring to patients who have sarcoma or a history of sarcoma. However, the presence of indeterminant nodules seen on chest CT scans can lead to a medical conundrum. Do these nodules represent metastatic disease or are they merely incidental findings? How worried should a patient be and how should the practitioner counsel the patient with regards to these indeterminant nodules? These questions can lead to anxiety for the patient and concerns about further testing and interventions for an incidental or equivocally positive finding [6].

When considering the relevance of indeterminant nodules on chest CT scans, some imaging features may be suggestive of metastasis. For osteosarcoma, as an example, such features include multiple nodules, size >5 mm, nodule calcifications, and change in the number or size during neoadjuvant chemotherapy [7, 8]. Imaging features of pulmonary metastases in Ewing's sarcoma are less well studied, but in general, those used for osteosarcoma, with the exception of calcification, are accepted [9]. Chondrosarcoma-concerning features include high grade and dedifferentiated chondrosarcoma histology, size >10 mm, bilateral nodules, and nodule calcifications [10]. None of these criteria have well-documented specificity, however. Indeed, benign pulmonary nodules are common across the general population [11]. Gould et al. conducted a retrospective observational study of adult members of an integrated healthcare system and found pulmonary nodules on 31% of all chest CT scans performed [12]. Even simple data regarding the incidence and relative risks of indeterminant nodules found on staging and surveillance chest CT scans are limited in the sarcoma medical literature.

The primary purpose of this study is to identify the behavior of indeterminant nodules found on initial chest CT staging in patients with primary sarcoma of bone without clear, evident metastatic disease on presentation. Using this cohort of patients, we investigate what percentage of patients with and without indeterminant nodules progress to metastatic disease and assess whether any patient-specific factors associate with the progression of indeterminant nodules to true metastatic disease. We also investigate the rate of development of indeterminant nodules during the surveillance period in patients with primary sarcoma of bone and the association of these indeterminant nodules with progression to metastatic disease.

## 2. Methods

After Institutional Review Board's approval, a retrospective review was performed of all patients presenting to a single National Cancer Institute Comprehensive Cancer Center with a primary sarcoma of bone between the years 2010 and 2020. Patients were included in the study if they met the following criteria: they were diagnosed with a primary sarcoma of bone proven through biopsy and histologically confirmed by a pathologist with specialty training in sarcoma; full initial staging imaging was performed per NCCN guidelines and no observable metastatic disease was evident; patients were at least 16 years of age; and preoperative and postoperative/post-treatment CT scans of the chest were available for review. Patients were excluded if patients did not have a confirmed diagnosis of a primary sarcoma of bone; they presented on initial staging with demonstrable metastatic disease as determined by biopsy of a lesion distant to the primary site of disease; they were less than 16 years of age; they did not have a pretreatment CT of the chest; or they did not have surveillance scans with post-treatment CT scans of the chest. Patients were included with follow-up CT scans as short as 2 months to capture early episodes of metastatic disease.

Patients were initially staged, treated, and followed in surveillance according to NCCN guidelines. Treatments for all osteosarcoma and Ewing sarcoma patients included chemotherapy and wide local resection; chondrosarcoma was treated with wide surgical resections; and chordoma was treated with radiation and wide surgical resection. Diagnoses grouped as “other” included entities such a leiomyosarcoma of bone, malignant fibrous histiocytoma of bone, and fibrosarcoma of bone; these were treated with osteosarcoma regimens and wide surgical resection.

During the study period, the intent of the hospital's sarcoma team was to complete surveillance for high-grade bone sarcomas by imaging each patient every 3 months for the first 2 years after surgery, every 6 months for the ensuing 3 years, and then yearly until 10 years after local control. Imaging consisted of CT scans of the chest, MRI of the anatomic location of the primary sarcoma, and additional imaging based on the histologic subtype of the sarcoma. Indeterminant nodules were recorded in radiology reports for the number and size of lesions and confirmed by over-read, as described by MacMahon et al. [13]. Once a nodule was identified on CT, it was followed on subsequent CT scans until it regressed or progressed to metastatic disease. Nodules were considered metastatic disease if they were histologically proven through core needle biopsy or clinical progression coinciding with a nonpulmonary biopsy, confirming metastasis. Patients were followed until the end of the surveillance period, to metastatic disease, to death, or to loss of follow-up.

Variables collected on each patient included age, gender, smoking status, sarcoma diagnosis, anatomic location of the primary tumor, treatments rendered, number and size of nodules identified on initial CT scan, number and size of nodules identified on post-treatment CT scans, progression vs. stability vs. regression of all pulmonary nodules, follow-up times, and event-free and overall survival.

### 2.1. Statistics

Descriptive statistics were collated for the cohort, including age, gender, histologic diagnosis, anatomic location, and presence of indeterminant nodules seen on CT scans, both at the initial presentation and the post-treatment surveillance time periods. Patients were initially grouped into 2 categories as follows: those with initial CT scans of the chest demonstrating indeterminant nodules and those with CT scans without indeterminant nodules. These groups were compared using Chi-square and Mann–Whitney *U* tests to determine if they differed in the dependent variables listed. These groups were then assessed for differences in the development of metastatic disease and overall survival. Chi-square and Mann–Whitney *U* tests were again used to assess for statistical significance in the development of metastatic disease; Kaplan–Meier survival curves were generated and compared with log-rank tests to assess differences in survival. The number and size of the indeterminant nodules were also recorded and assessed for association with the development of metastatic disease. Cox regression analysis was performed, and hazard ratios were generated to determine the relative influence and effect of age, histologic diagnosis, anatomic location, size of the largest nodules, and number of nodules on the development of metastatic disease.

Follow-up groups were then created by categorizing those who developed indeterminant nodules during surveillance CT scans of the chest and those who did not develop indeterminant nodules. Variables of age, gender, histologic diagnosis, anatomic location, and presence of indeterminant nodules seen on the initial CT scan were then compared between the two groups using Chi-square and Mann–Whitney *U* tests. A *p* value of 0.05 was set for statistical significance.

## 3. Results

**Figure 1:**
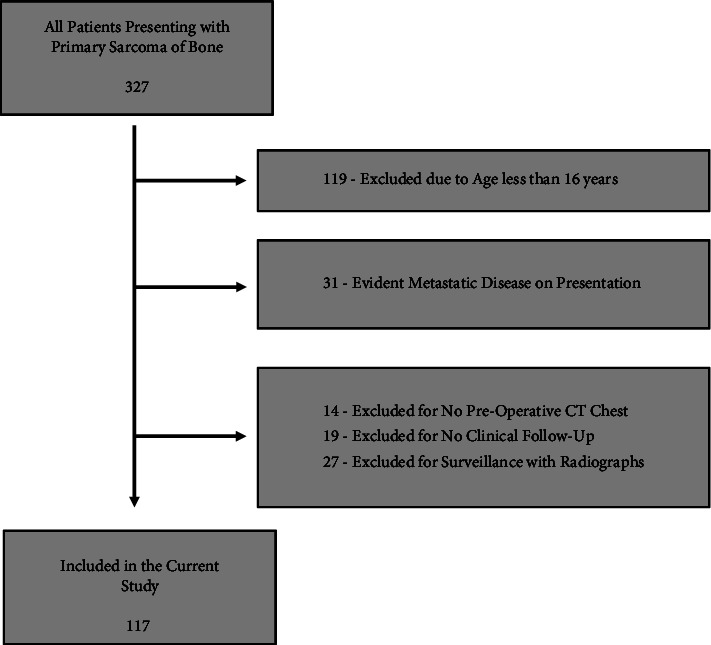
Inclusion and exclusion of patients.

A total of 327 patients presented to the Sarcoma Team with a diagnosis of primary sarcoma of bone during the study period. After exclusion for age, evident metastatic disease on presentation, absence of preoperative CT scan of the chest, lack of clinical follow-up, and surveillance with chest radiographs instead of CT scans, 117 patients met inclusion criteria (Figure 1).

The average age of the patients in the included cohort was 48.6 years (range: 16–81 years). Follow-up averaged 38.2 months, ranging from 2 to 192 months. There was a preponderance of male patients (59%, *n* = 69); 30.8% (*n* = 36) of the cohort had a smoking history. Chondrosarcoma was the most common diagnosis, followed by osteosarcoma. All patients who had a diagnosis of osteosarcoma, Ewing sarcoma, and “others” were treated with a regimen of chemotherapy and wide surgical resection. All chondrosarcomas were treated with wide surgical resection alone. Three of the four chordomas were treated with radiation and surgery, while 1 of the 4 was treated with surgery alone. The vast majority of patients had high-grade sarcomas (93.2%, *n* = 109). Eight low-grade sarcoma cases were included, as they had sufficient clinical or radiographic concern to warrant high-grade surveillance, as deemed by the oncologic team at the time. The lower extremity was the most common anatomic site (41%, *n* = 48), followed by the pelvis (29.9%, *n* = 35) and then the upper extremity (17.9%, *n* = 21).

**Figure 2:**
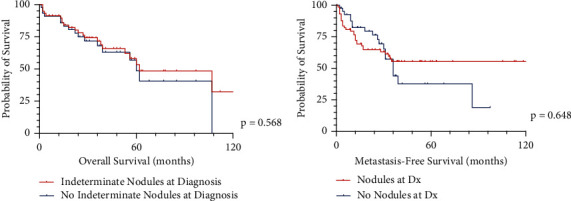
Kaplan–Meier curve: (a) overall survival and (b) metastasis-free survival in patients with indeterminant nodules on initial presentation versus those that did not have indeterminant nodules on initial presentation.

Of the entire cohort, 62.4% (*n* = 73) of the group had indeterminant nodules on presurgical CT scans of the chest. When comparing those with indeterminant nodules on initial CT scans of the chest to those without indeterminant nodules, there were no differences in the percentages of each group regarding gender, smoking status, diagnosis, grade of tumor, or anatomic location (Table 1). The group that had indeterminant nodules on initial CT scans of the chest went on to develop metastatic disease in 41.1% of the cases, in comparison to 43.2% for those who presented without any nodules on initial chest CT scan (*p*=0.825). Time to metastatic disease averaged 13.5 months from surgery for the group with indeterminant nodules and 24.9 months for the group without indeterminant chest nodules (*p*=0.008).  Figure 2 presents a Kaplan–Meier curve for the overall survival and the metastasis-free survival between the two groups, censoring for losses to the follow-up (*p*=0.568 and 0.648, respectfully).

Of the 73 patients presenting with indeterminant nodules at time of initial staging, 41.1% (*n* = 30) went on to develop metastatic disease (Table 2). When comparing the group with initial indeterminant nodules that developed metastatic disease to those with initial indeterminant nodules that did not develop metastatic disease, there were no significant differences between the groups with regards to age, gender, smoking history, sarcoma diagnosis, grade of tumor, anatomic location of the primary site of disease, size of the pulmonary nodules, or the number of indeterminant nodules seen at initial disease presentation. There was a difference between these two groups in total months of postoperative follow-up. Those without metastatic disease were followed for a mean of 53.7 months (range: 2–193 months), while those who eventually developed metastatic disease were followed for a mean of 22.9 months (range: 2–107 months) (*p* < 0.001). This difference is attributed to survival, as the median overall survival group that developed metastatic disease was 24.0 months (95% CI: 2.07–45.93 months).

Fifty eight of the 117 total patients (49.6%) developed indeterminant chest nodules on follow-up surveillance CT scans, while 59 patients did not (50.4%). These two groups did not demonstrate significant differences in age, gender, smoking history, sarcoma diagnosis, grade of tumor, or anatomic location of the primary site of disease (Table 3). When considering the presence of indeterminant chest nodules at the time of initial disease presentation in relation to the development of new nodules during the surveillance follow-up, 43.1% of the patients who developed new nodules during surveillance had indeterminant nodules at the time of initial presentation, while 81.4% of patients who did not develop nodules on the follow-up did have indeterminant nodules at the time of initial presentation (*p* < 0.001). There were no differences between the group that developed indeterminant chest nodules in surveillance and the group that did not develop new nodules in surveillance, when considering the size and number of those nodules observed at the time of initial disease presentation (*p*=0.253 and 0.059, respectively). Whether a patient developed indeterminant nodules on chest CT scans during the surveillance period had a significant correlation with the likelihood of developing metastatic disease. Thirty two of the 58 (55.2%) patients who developed indeterminant nodules on surveillance chest CT scans went on to develop metastatic disease at an average of 21.2 months after local control surgery. Seventeen of the 59 patients (28.8%) who did not develop any new lung nodules on follow-up CT scans still went on to be diagnosed with metastatic disease at an average of 11.4 months. The time to metastasis between these groups (21.2 months vs. 11.4 months) was statistically different (*p*=0.033). All 17 of these patients who were eventually diagnosed with metastatic disease but did not develop any new lesions on surveillance chest CT scans had indeterminant nodules at initial presentation, which progressed into the diagnosed metastatic disease. Of note, once metastatic disease was documented, we did not continue to follow any subsequent CT scans to determine if new nodules would then develop, as diagnosis of metastatic disease was the endpoint of interest for this study.

Table 4 describes the group of patients that developed indeterminant nodules on surveillance chest CT scans, comparing the subgroups that developed metastatic disease to those that did not. These subgroups did not differ in age, gender, follow-up time, smoking history, sarcoma diagnosis, grade of tumor, anatomic location of the primary sarcoma, or the presence of indeterminant chest nodules found on preoperative CT scan. Size of the largest nodule that developed on surveillance chest CT scan and the number of nodules that developed were significantly different between the group that ultimately developed metastatic disease and the group that did not (*p*=0.014 and 0.021 respectively). Those that developed metastatic disease were more likely to have larger nodules on their surveillance scans, as the group that did not develop metastatic disease only developed nodules larger than 5 mm 26.9% of the time, while the group that did progress to metastatic disease had nodules larger than 5 mm 65.7% of the time (*p*=0.014). Likewise, patients that progressed to metastatic disease were more likely to develop multiple chest nodules while those that did not progress to metastatic disease were more likely to develop a solitary nodule on chest surveillance (*p*=0.021).

The results of the regression analysis are presented in Table 5. For this cohort of patients with primary sarcoma of bone not presenting with metastatic disease, age was not found to associate with the development of metastatic disease nor did gender or histologic diagnosis. Anatomic location was assessed with the lower extremity site as the standard, resulting in no significant associations with anatomic location to the development of metastatic disease. The presence of indeterminant nodules on preoperative chest CT did not demonstrate a predictive influence on the development of metastatic disease (HR: 0.981 and CI: 0.493–1.955). The development of indeterminant nodules on the follow-up trended towards significance in regard to associating with metastatic disease, but the confidence interval crossed one (HR: 2.008, CI: 0.997–4.041, and *p*=0.051).

**Figure 3:**
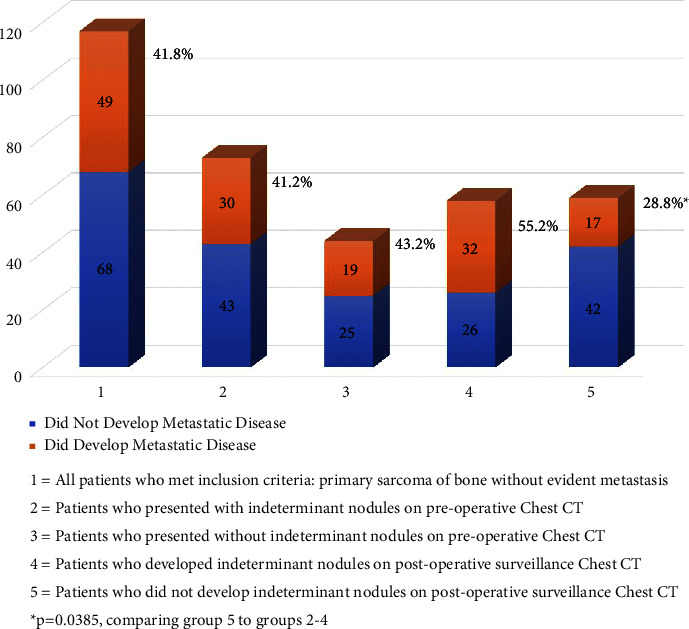
Development of metastatic disease based on the presence or development of indeterminant chest nodules.

Figure 3 presents a bar graph depicting the relative likelihood of developing metastatic disease in patients presenting with nonmetastatic primary sarcoma of bone based on the presence or absence of indeterminant chest nodules on initial staging or on surveillance chest CT scans. When compared to the cohort as a whole, there was no difference in the eventual development of metastatic disease, whether a patient presented with indeterminant nodules or not on the initial chest CT scan. There was a significant difference in metastasis development when comparing the group that did not develop nodules on the follow-up CT compared to the group that did develop nodules on surveillance CT scans (28.5% vs. 55.2%, respectively, *p*=0.039).

**Figure 4:**
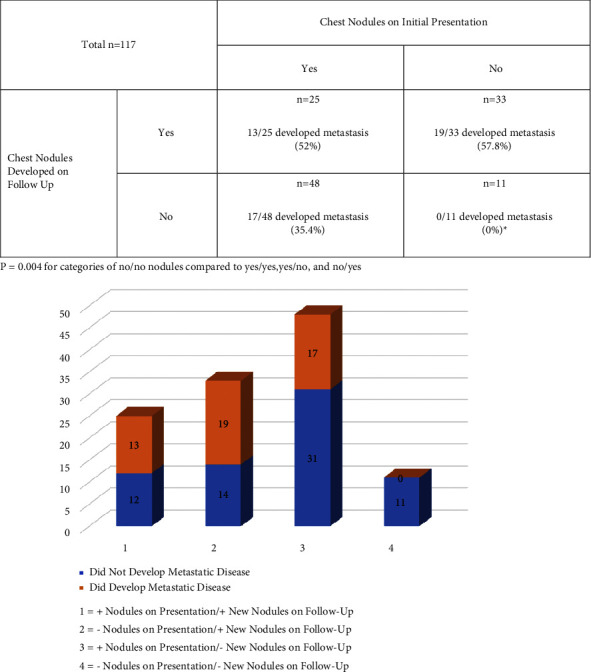
Development of metastatic disease in patients with primary sarcoma of bone grouped by the presence and development of pulmonary nodules during cancer staging.

Figure 4 presents the cohort divided into a Punnett square based on the presence of indeterminant nodules at the time of initial staging (yes/no) and whether the patient developed indeterminant nodules on surveillance scans (yes/no). The only combination that resulted in a statistically different rate of metastasis was the group that had no indeterminant nodules at presentation and developed no pulmonary nodules on the follow-up. This subset of patients (*n* = 11 of 117) had a 0% occurrence of metastatic disease (*p*=0.004).

## 4. Discussion

The clinical significance of indeterminant pulmonary nodules seen during the workup and subsequent surveillance of bone sarcoma patients remains uncertain. Suspicion of any pulmonary nodule must remain high, given the proclivity of bone sarcoma to metastasize to the lung [14–16]. However, clinical practice informs us that not all indeterminant pulmonary nodules portend metastatic disease. In their retrospective cohort study of patients with bone or soft tissue sarcomas, Mayo et al. found that 33% of their patients presented with indeterminate pulmonary nodules, but only 31% of that subset developed true metastatic disease [17]. The primary aim of this study was to determine the incidence of pulmonary nodules in a group of patients presenting with presumptive localized primary sarcoma of bone and to assess if there were any dependent variables that associated with the subsequent development of metastatic disease. In this cohort of patients, 62.4% of the patients presenting with a nonmetastatic primary sarcoma of bone had at least one indeterminant pulmonary nodule on the initial staging CT scan of the chest. When comparing the group that had indeterminant pulmonary nodules on pretreatment CT scans to those that had no nodules, the rates of eventual development of metastatic disease were no different (41.1% vs. 43.2%, respectively; *p*=0.825). In addition, there were no dependent variables that we studied that associated with the progression of these nodules to metastatic disease, including the number and size of the nodules. This suggests that at the baseline, the presence of indeterminate pulmonary nodules on initial chest staging do not reflect or predict a patient's likelihood of developing metastatic disease in the future. This is contrary to the findings of Rissing et al. that found nodules 5 mm or greater on initial staging CT scans were predictive of the development of true metastatic disease [18].

The secondary aim of this study was to determine the incidence of indeterminant pulmonary nodule development on surveillance CT scans and to determine if there were dependent variables that associated with metastatic disease progression in this subset of patients. Nearly half of the cohort developed an indeterminant nodule during the post-treatment surveillance period (49.6%). Of this subset, only slightly more than half eventually progressed to metastatic disease (55.2%). When considering the development of indeterminant nodules on surveillance chest imaging and the likelihood that these nodules will progress to evident metastatic disease, the size and number of the nodules appear to be important. Patients that had larger nodules were more likely to progress to metastatic disease and patients that had more nodules were likewise more apt to progress to metastatic disease. This is similar to prior studies that have identified size and number to be important factors in development of metastatic disease. Nakamura et al. found that pulmonary nodules that exceeded 5 mm were predictive of being true metastatic disease in their studied cohort [19].

When taken together, the presence of indeterminant nodules at the time of disease presentation and development of indeterminant nodules during surveillance, there remained little predictive value in who would eventually progress to metastatic disease. For instance, 25 patients had both chest nodules at the time of presentation and developed pulmonary nodules during surveillance; only 13 (52.0%) of these 25 went on to develop metastatic disease. In this data set of adult patients with presumptive localized primary sarcoma of bone, the only circumstance, in relation to pulmonary nodules, that strongly predicted whether a patient would develop metastatic disease or not was the case of having no nodules at the time of presentation and no nodules develop on surveillance. This circumstance was met in only 9.4% (*n* = 11) of the cohort. None of these patients had metastatic disease at the time of final follow-up (mean: 35.6 months).

There are several limitations to this study. First, the retrospective nature of the study limits the data to that which were collected and available for review. Sixty patients were excluded due to absence of preoperative chest CT (*n* = 14), no clinical follow-up (*n* = 19), or lack of surveillance CT scans (*n* = 27). While that number amounts to 18.3% of the total cohort, it actually represents 33.9% of the cohort that would have met inclusion criteria once patients were excluded for age (*n* = 119) and clearly metastatic disease at the time of presentation (*n* = 31). This is a substantial percentage, and the unknown effects of these missing data must be acknowledged. Second, we excluded pediatric patients from the assessment, limiting the cohort to adults. This decreases the generalizability of the results and likely obscured the effect of age on metastatic progression. We felt that the pediatric and adult populations represent distinct cohorts and did not want to mix these populations, as they may have a different likelihood of having incidentally found pulmonary nodules. Indeed, Kaste et al. found the number of nodules and bilateral nodules on initial staging CT scans to be predictive of true metastatic disease in their cohort of pediatric patients with osteosarcoma [20]. As such, the effect of indeterminant pulmonary nodules on the prognosis of pediatric patients with bone sarcoma should be considered and studied of its own accord. Third, this cohort of patients resided in a predominantly arid geography of the Mountain West region of the United States. This may reflect an environmental influence, making the likelihood of noncancerous pulmonary nodule development more likely. Other geographies, with less (or more) airborne particulates, may have a lower (or higher) incidence of benign nodule formation. Suggestive of this, Mayo et al. [17] found incidental pulmonary nodules in 33% of their sarcoma patients at the time of initial staging compared to 62.4% in the current study. Finally, our cohort is small. With 117 subjects meeting inclusion, the power to detect minor differences is limited.

Despite the limitations in the study design and execution, we believe this investigation brings needed clarity to the issue of staging and surveillance of patients with primary sarcomas of bone. Chest imaging is crucial, as the clinical implications of evident metastatic disease are profound and immediate. Treatment strategies often change based on the diagnosis of localized versus metastatic disease. Certainly, having no nodules on initial staging is a moment of relief for patients dealing with a very troublesome and anxiety provoking diagnosis, but these data suggest that this is no reason to lose vigilance, as the rate of subsequent metastasis is the same as those with indeterminant pulmonary nodules. Likewise, for those with indeterminant nodules, there is reason to hope, as 58.9% of this group did not develop metastases on clinical surveillance. With this in mind, treatment decisions should not be made based on indeterminant nodules, aside from continued vigilance, and patients should be informed of the “grey areas” that current medical practice resides, with regards to pulmonary nodules that can be seen but not biopsied.

## 5. Conclusion

Indeterminant pulmonary nodules remain a prognostic conundrum for patients with primary sarcomas of bone. In this series of adult patients with localized primary sarcoma of bone, 62.4% of the patients had indeterminant nodules on initial staging CT chest imaging. Presence of these nodules did not predict progression to metastatic disease from the group that had no nodules. None of the dependent variables studied (age, gender, histology, anatomic location, number of nodules, and size of nodules) had an association or predictive impact on whether a patient with an indeterminant nodule at the time of disease presentation would progress to metastatic disease. Development of pulmonary nodules after local control did significantly correlate with the subsequent development of metastatic disease, however, with the size and number of nodules demonstrating a significant impact on the likelihood of developing metastatic disease.

## Figures and Tables

**Table 1:** Descriptive data of the initial, preoperative CT chest findings in patients presenting with a primary sarcoma of the bone without known metastatic disease at the time of diagnosis.

		All patients	Patients presenting with indeterminant nodules on initial/preoperative CT chest	Patients presenting without nodules on initial/preoperative CT chest	*p* value^*∗*^
*N*		117	73	44	

Age (years)		48.6 (16–81)	50 (19–81)	46.2 (16–76)	0.281

Follow-up (months)		38.2 (2–192)	40.9 (2–192)	33.8 (2–107)	0.432

Gender	Male	69	41 (56.2%)	28 (63.6%)	0.426
	Female	48	32 (43.8%)	16 (36.4%)	

Smoking history	Never	70	42 (57.5%)	28 (63.6%)	0.543
	+ Smoking history	36	25 (34.2%)	11 (25.0%)	
	Unknown	11	6 (8.2%)	5 (11.4%)	

Diagnosis	Osteosarcoma	36	24 (32.9%)	12 (27.3%)	0.886
	Chondrosarcoma	49	30 (41.1%)	19 (43.2%)	
	Ewing sarcoma	8	4 (5.5%)	4 (9.1%)	
	Chordoma	4	2 (2.7%)	2 (4.5%)	
	Others	20	13 (17.8%)	7 (15.9%)	

Grade	Low	8	5 (6.8%)	3 (6.8%)	0.995
	High	109	68 (93.2%)	41 (93.2%)	

Anatomic location	Upper extremity	21	10 (13.7%)	11 (25.0%)	0.476
	Lower extremity	48	32 (43.8%)	16 (36.4%)	
	Spine/chest wall	13	8 (11.0%)	5 (11.4%)	
	Pelvis	35	23 (31.5%)	12 (27.3%)	

Progressed to metastatic disease	Yes	49	30 (41.1%)	19 (43.2%)	0.825
	No	68	43 (58.9%)	25 (56.8%)	

Time to metastatic disease (months)		16.7 (2–86)	11.6 (2–40)	24.6 (2–86)	0.008

^*∗*^ *p* value: comparison of patients presenting with indeterminant nodules vs. presenting without nodules for each considered variable, performed with Chi-square and Mann–Whitney *U* tests.

**Table 2:** Patients presenting with primary sarcoma of bone without evident metastatic disease on initial staging but with indeterminant nodules on CT scan of the chest: descriptive data of patients; the subgroups that did and did not develop metastatic disease, along with comparison between the groups that developed metastatic disease vs. those that did not.

		All patients presenting with indeterminant nodules on initial CT chest scan	Patients who progressed to metastatic disease	Patients who did not progress to metastatic disease	*p* value^*∗*^
*N*		73	30	43	

Age (years)		50 (19–81)	50.9 (19–81)	49.5 (24–79)	0.814

Follow-up (months)		40.9 (2–192)	22.9 (2–78)	53.7 (8–192)	<0.001

Gender	Male	41 (56.2%)	16 (53.3%)	25 (58.1%)	0.684
	Female	32 (43.8%)	14 (46.7%)	18 (41.9%)	

Smoking history	Never	42 (57.5%)	17 (56.7%)	25 (58.1%)	0.898
	+Smoking history	25 (34.2%)	10 (33.3%)	15 (34.9%)	
	Unknown	6 (8.2%)	3 (10.0%)	3 (7.0%)	

Diagnosis	Osteosarcoma	24 (32.9%)	9 (30.0%)	15 (34.9%)	0.637
	Chondrosarcoma	30 (41.1%)	12 (40.0%)	18 (41.9%)	
	Ewing sarcoma	4 (5.5%)	2 (6.7%)	2 (4.7%)	
	Chordoma	2 (2.7%)	0 (0%)	2 (4.7%)	
	Others	13 (17.8%)	7 (23.3%)	6 (14.0%)	

Grade	Low	5 (6.8%)	1 (3.3%)	4 (9.3%)	0.321
	High	68 (93.2%)	29 (96.7%)	39 (90.7%)	

Anatomic location	Upper extremity	10 (13.7%)	3 (10.0%)	7 (16.3%)	0.805
	Lower extremity	32 (43.8%)	13 (43.3%)	19 (44.2%)	
	Spine/chest wall	8 (11.0%)	3 (10.0%)	5 (11.6%)	
	Pelvis	23 (31.5%)	11 (36.7%)	12 (27.9%)	

Size of the largest indeterminant nodule	1–5 mm	57 (78.1%)	22 (73.3%)	35 (81.4%)	0.615
	6–9 mm	11 (15.1%)	6 (20.0%)	5 (11.6%)	
	10+ mm	5 (6.8%)	2 (6.7%)	3 (7.0%)	

Number of nodules	1	20 (27.4%)	7 (23.3%)	13 (30.2%)	0.712
	2	21 (28.8%)	8 (26.7%)	13 (30.2%)	
	3	18 (24.7%)	8 (26.7%)	10 (23.3%)	
	4	6 (8.2%)	4 (13.3%)	2 (4.7%)	
	5+	8 (11.0%)	3 (10.0%)	5 (11.6%)	

^*∗*^ *p* value: comparison of patients presenting with indeterminant nodules who went on to develop metastatic disease vs. those who did not develop metastatic disease, performed with Chi-square and Mann–Whitney *U* tests.

**Table 3:** Development of indeterminant chest CT nodules in patients with primary sarcoma of the bone without evident metastatic disease on presentation.

		Patients who developed indeterminant nodules on follow-up CT scans of the chest	Patients who did not develop indeterminant nodules on follow-upCT scans of the chest	*p* value^*∗*^
*N*		58	59	

Age (years)		49.6 (16–81)	47.7 (16–77)	0.591

Follow-up (months)		40.8 (2–192)	35.9 (1–128)	0.189

Gender	Male	34 (58.6%)	35 (59.3%)	0.939
	Female	24 (41.4%)	24 (40.7%)	

Smoking history	Never	38 (65.5%)	32 (54.2%)	0.450
	+Smoking history	15 (25.9%)	21 (35.6%)	
	Unknown	5 (8.6%)	6 (10.2%)	

Diagnosis	Osteosarcoma	14 (24.1%)	22 (37.3%)	0.123
	Chondrosarcoma	23 (39.7%)	26 (44.1%)	
	Ewing sarcoma	5 (8.6%)	3 (5.1%)	
	Chordoma	4 (6.9%)	0 (0%)	
	Others	12 (20.7%)	8 (13.6%)	

Grade	Low	3 (5.2%)	5 (8.5%)	0.479
	High	55 (94.8%)	54 (91.5%)	

Anatomic location	Upper extremity	11 (19.0%)	10 (16.9%)	0.619
	Lower extremity	22 (37.9%)	26 (44.1%)	
	Spine/chest wall	5 (8.6%)	8 (13.6%)	
	Pelvis	20 (34.5%)	15 (25.4%)	

Presence of indeterminant nodules on preoperative CT chest	Yes	25 (43.1%)	48 (81.4%)	<0.001
	No	33 (56.9%)	11 (18.6%)	

If present, size of largest nodule on preoperative CT chest	1–5 mm	20 (80.0%)	37 (77.1%)	0.253
	6–9 mm	2 (8.0%)	9 (18.8%)	
	10+ mm	3 (12.0%)	2 (4.2%)	

If present, number of nodules each patient had at the time of preoperative CT chest	1	11 (44.0%)	9 (18.8%)	0.059
	2	8 (32.0%)	13 (27.1%)	
	3	5 (20.0%)	13 (27.1%)	
	4	0 (0%)	6 (12.5%)	
	5+	1 (4.0%)	7 (14.6%)	

Progressed to metastatic disease	Yes	32 (55.2%)	17 (28.8%)	0.004
	No	26 (44.8%)	42 (71.2%)	

Time to metastatic disease (months)		20.0 (2–86)	10.3 (2–35)	0.033

^*∗*^ *p* value: comparison of patients presenting with indeterminant nodules who went on to develop metastatic disease vs. those who did not develop metastatic disease, performed with Chi-square and Mann–Whitney *U* tests.

**Table 4:** Patients presenting with primary sarcoma of the bone without evident metastatic disease who subsequently developed indeterminant nodules on CT scan of the chest: comparison between the subgroup that developed metastatic disease vs. those that did not.

		Patients who developed indeterminant nodules on follow-up CT scans of the chest who then developed metastatic disease	Patients who developed indeterminant nodules on follow-up CT scans of the chest but did not develop metastatic disease	*p* value^*∗*^
*N*		32	26	

Age (years)		48.3 (19–81)	51.1 (16–79)	0.622

Follow-up (months)		37.3 (2–107)	45.2 (2–192)	0.579

Gender	Male	19 (59.4%)	15 (57.7%)	0.897
	Female	13 (40.6%)	11 (42.3%)	

Smoking history	Never	21 (65.6%)	17 (65.4%)	0.967
	+Smoking history	8 (25.0%)	7 (26.9%)	
	Unknown	3 (9.4%)	2 (7.7%)	

Diagnosis	Osteosarcoma	8 (25.0%)	6 (23.1%)	0.761
	Chondrosarcoma	11 (34.4%)	12 (46.2%)	
	Ewing sarcoma	4 (12.5%)	1 (3.8%)	
	Chordoma	2 (6.3%)	2 (7.7%)	
	Others	7 (21.9%)	5 (19.2%)	

Grade	Low	1 (3.1%)	2 (7.7%)	0.435
	High	31 (96.9%)	24 (92.3%)	

Anatomic location	Upper extremity	3 (9.4%)	8 (30.8%)	0.219
	Lower extremity	13 (40.6%)	9 (34.6%)	
	Spine/Chest wall	3 (9.4%)	2 (7.7%)	
	Pelvis	13 (40.6%)	7 (26.9%)	

Presence of indeterminant nodules on preoperative CT chest	Yes	13 (40.6%)	12 (46.2%)	0.672
	No	19 (59.4%)	14 (53.8%)	

Size of largest nodule on preoperative CT chest	1–5 mm	12 (92.3%)	8 (66.7%)	0.212
	6–9 mm	0 (0%)	2 (16.7%)	
	10+ mm	1 (7.7%)	2 (16.7%)	

Time to development of indeterminant nodule from surgery (months)		15.5 (1–84)	14.3 (1–48)	0.650

Size of largest nodule each patient developed on surveillance CT chest	1–5 mm	11 (34.4%)	19 (73.1%)	0.014
	6–9 mm	11 (34.4%)	5 (19.2%)	
	10+ mm	10 (31.3%)	2 (7.7%)^a^	

Number of nodules each patient developed on surveillance CT chest	1	9 (28.1%)	17 (65.4%)	0.021
	2	6 (18.8%)	4 (15.4%)	
	3	5 (15.6%)	4 (15.4%)	
	4	4 (12.5%)	1 (3.8%)	
	5+	8 (25.0%)	0 (0%)	

^*∗*^ *p* value: comparison of patients presenting with indeterminant nodules who went on to develop metastatic disease vs. those who did not develop metastatic disease, performed with Chi-square and Mann–Whitney *U* tests.

**Table 5:** Regression analysis for metastasis-free survival for patients with primary sarcoma of the bone without evident metastatic disease on presentation.

		Hazard ratio	CI	*p* value
Age		1.012	0.994–1.030	0.191

Gender	Male	1.089	0.587–2.020	0.787
	Female			

Diagnosis	Osteosarcoma	0.739	0.354–1.542	0.420
	Chondrosarcoma	0.909	0.274–3.011	0.876
	Ewing sarcoma	0.333	0.066–1.679	0.183
	Chordoma	1.406	0.626–3.161	0.409
	Others			

Anatomic location	Lower extremity	0.366	0.123–1.091	0.071
	Upper extremity	0.870	0.286–2.643	0.806
	Spine/chest wall	1.412	0.729–2.733	0.307
	Pelvis			

Presence of indeterminant nodules on preoperative CT chest	Yes	0.981	0.493–1.955	0.958
	No			

Development of indeterminant nodules on surveillance CT chest	Yes	2.008	0.997–4.041	0.051
	No			

## Data Availability

The data used to support the findings of this study are held secure in an institutional database and are available from the corresponding author upon reasonable request.

## References

[1] Friebele J. C., Peck J., Pan X., Abdel-Rasoul M., Mayerson J. L. (2015). Osteosarcoma: a meta-analysis and review of the literature. *American Journal of Orthopedics*.

[2] Ye C., Dai M., Zhang B. (2019). Risk factors for metastasis at initial diagnosis with ewing sarcoma. *Frontiers in Oncology*.

[3] Marulli G., Mammana M., Comacchio G., Rea F. (2017). Survival and prognostic factors following pulmonary metastasectomy for sarcoma. *Journal of Thoracic Disease*.

[4] Biermann J., Hirbe A. (2023). Bone cancer version 3.2023. https://www.webmd.com/cancer/bone-tumors.

[5] Ost D., Fein A. M., Feinsilver S. H. (2003). The solitary pulmonary nodule. *New England Journal of Medicine*.

[6] Tepper S. C., Holten A. K., Jeffe D. B. (2022). Examining patient perspectives on sarcoma surveillance: the Sarcoma Surveillance Survey. *Surgical oncology*.

[7] Picci P., Vanel D., Briccoli A. (2001). Computed tomography of pulmonary metastases from osteosarcoma: the less poor technique. A study of 51 patients with histological correlation. *Annals of Oncology*.

[8] Ciccarese F., Bazzocchi A., Ciminari R. (2015). The many faces of pulmonary metastases of osteosarcoma: retrospective study on 283 lesions submitted to surgery. *European Journal of Radiology*.

[9] Saifuddin A., Baig M. S., Dalal P., Strauss S. J. (2021). The diagnosis of pulmonary metastases on chest computed tomography in primary bone sarcoma and musculoskeletal soft tissue sarcoma. *British Journal of Radiology*.

[10] McLoughlin E., Davies A. M., Iqbal A., James S. L., Botchu R. (2020). The diagnostic significance of pulmonary nodules on CT thorax in chondrosarcoma of bone. *Clinical Radiology*.

[11] Tanner N. T., Aggarwal J., Gould M. K. (2015). Management of pulmonary nodules by community pulmonologists: a multicenter observational study. *Chest*.

[12] Gould M. K., Tang T., Liu I. L. A. (2015). Recent trends in the identification of incidental pulmonary nodules. *American Journal of Respiratory and Critical Care Medicine*.

[13] MacMahon H., Naidich D. P., Goo J. M. (2017). Guidelines for management of incidental pulmonary nodules detected on CT images: from the Fleischner Society. *Radiology*.

[14] Ladenstein R., Pötschger U., Le Deley M. C. (2010). Primary disseminated multifocal Ewing sarcoma: results of the Euro-EWING 99 trial. *Journal of Clinical Oncology*.

[15] Gelderblom H., Jinks R. C., Sydes M. (2011). Survival after recurrent osteosarcoma: data from 3 European Osteosarcoma Intergroup (EOI) randomized controlled trials. *European Journal of Cancer*.

[16] Ozaki T., Hillmann A., Lindner N., Blasius S., Winkelmann W. (1996). Metastasis of chondrosarcoma. *Journal of Cancer Research and Clinical Oncology*.

[17] Mayo Z., Kennedy S., Gao Y., Miller B. J. (2019). What is the clinical importance of incidental findings on staging CT scans in patients with sarcoma?. *Clinical Orthopaedics and Related Research*.

[18] Rissing S., Rougraff B. T., Davis K. (2007). Indeterminate pulmonary nodules in patients with sarcoma affect survival. *Clinical Orthopaedics and Related Research*.

[19] Nakamura T., Matsumine A., Rui N. (2009). Management of small pulmonary nodules in patients with sarcoma. *Clinical & Experimental Metastasis*.

[20] Kaste S. C., Charles B., Pratt A. M. C., Jones‐Wallace D. J., Rao B. N. (1999). Metastases detected at the time of diagnosis of primary pediatric extremity osteosarcoma at diagnosis: imaging features. *Cancer*.

